# The role of the tumor microbe microenvironment in the tumor immune microenvironment: bystander, activator, or inhibitor?

**DOI:** 10.1186/s13046-021-02128-w

**Published:** 2021-10-16

**Authors:** Jiayao Ma, Lingjuan Huang, Die Hu, Shan Zeng, Ying Han, Hong Shen

**Affiliations:** 1 Department of Oncology, Xiangya Hospital, Central South University, Changsha, 410008 Hunan China; 2 Department of Dermatology, Xiangya Hospital, Central South University, Changsha, 410008 Hunan China; 3 Xiangya Medical College, Central South University, Changsha, 410013 Hunan China; 4 Key Laboratory for Molecular Radiation Oncology of Hunan Province, Xiangya Hospital, Central South University, Changsha, 410008 Hunan China; 5 National Clinical Research Center for Geriatric Disorders, Xiangya Hospital, Central South University, Changsha, Hunan 410008 P.R. China

**Keywords:** Tumor microenvironment, Microbiome, Tumor immune microenvironment, Immune checkpoint inhibitor, Therapeutic target

## Abstract

The efficacy of cancer immunotherapy largely depends on the tumor microenvironment, especially the tumor immune microenvironment. Emerging studies have claimed that microbes reside within tumor cells and immune cells, suggesting that these microbes can impact the state of the tumor immune microenvironment. For the first time, this review delineates the landscape of intra-tumoral microbes and their products, herein defined as the tumor microbe microenvironment. The role of the tumor microbe microenvironment in the tumor immune microenvironment is multifaceted: either as an immune activator, inhibitor, or bystander. The underlying mechanisms include: (I) the presentation of microbial antigens by cancer cells and immune cells, (II) microbial antigens mimicry shared with tumor antigens, (III) microbe-induced immunogenic cell death, (IV) microbial adjuvanticity mediated by pattern recognition receptors, (V) microbe-derived metabolites, and (VI) microbial stimulation of inhibitory checkpoints. The review further suggests the use of potential modulation strategies of the tumor microbe microenvironment to enhance the efficacy and reduce the adverse effects of checkpoint inhibitors. Lastly, the review highlights some critical questions awaiting to be answered in this field and provides possible solutions. Overall, the tumor microbe microenvironment modulates the tumor immune microenvironment, making it a potential target for improving immunotherapy. It is a novel field facing major challenges and deserves further exploration.

## Background

The human body accommodates trillions of microbes, some of which contribute to carcinogenesis or anticancer response [1–3]. The therapeutic effects of the microbes on solid tumors were documented over one century ago when doctor William Coley injected an extracted mixture of infectious bacteria directly into the tumors of the patients and reported a miraculous tumor regression [4, 5]. However, the experiments of Coley were poorly duplicatable in the following years. And the mechanisms underlying how the microbial infection elicited the tumor regression remained unclear [6]. Exogenous biocontamination and culture-dependent methods were the major limitations in microbial studies at that time [7–9].

The past decades have seen rapid advances in DNA sequencing technologies, which have liberated microbial identification from cultivation [8, 9]. For example, analysis of blood and tumor tissue from the Cancer Genome Atlas (TCGA) has allowed the identification of the tumor-type specific microbiome [10]. A large-scale study employing multiple identification strategies and strict contamination controls has further detailed the microbiome specific for cancer type and subtype [11]. This study has identified the bacteria and bacterial structures that reside within cancer cells and tumor-infiltrating immune cells, suggesting that the microbes have the potential to impact the tumor immune microenvironment (TIME) -- a determinant for the efficacy of immune checkpoint inhibitors (ICIs) [11–16]. Tumors dominated by the taxon of the *Gostridium* have a better response to ICIs compared to those dominated by the taxon of the *Cardnerella raginalis*, suggesting the microbes have the potential to modulate or predict ICIs efficacy [11]. However, the landscape of the intra-tumoral microbes remains to be characterized. And there is no framework to explain the mechanisms by which the intra-tumoral microbes influence the TIME.

This review aims to delineate the landscape of intra-tumoral microbes and the mechanisms underlying their roles in the TIME. It also aims to outline the therapeutic strategies which may modulate the tumor microbe microenvironment and influence ICIs efficacy. Building frameworks for such evidence will provide a novel perspective on precision medicine and combinatorial options to immunotherapy.

### The tumor immune microenvironment (TIME)

**Fig. 1:**
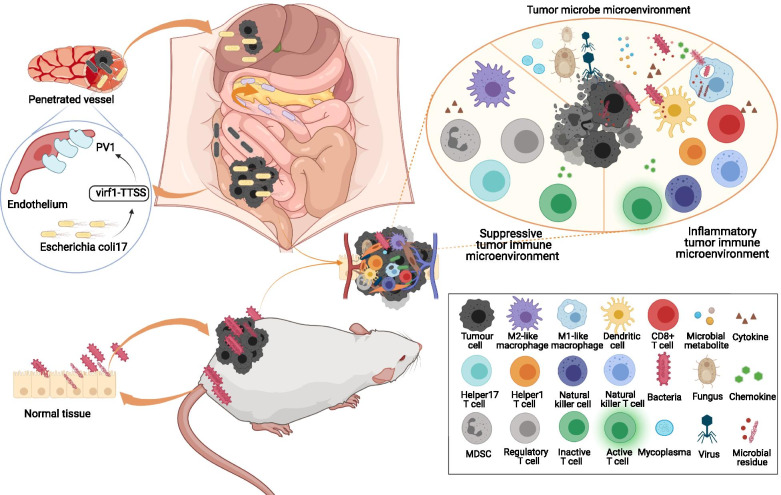
The landscape of the tumor microbe microenvironment and the tumor immune microenvironment. Microbes, microbial residues, and microbial metabolites reside within tumors, herein defined as the tumor microbe microenvironment. Intra-tumoral microbes come from the tissue where tumors initiate or from distal organs or metastasis through penetrated vessels. The major cells types in tumor immune microenvironment are divided into two categories: One is inflammatory and includes active CD8^+^ T cell, helper 1 T cell, dendritic cell, natural killer cell, natural killer T cell, M1-like macrophage, and so on; the other one is immunosuppressive and includes M2-like macrophage, regulatory T cell, helper 17 T cell, MDSC, inactive CD8^+^ T cell and so on. Abbreviations: CD = cluster of differentiation, MDSC = myeloid-derived immunosuppressive cell

The tumor immune microenvironment (TIME) refers to the microenvironment formed by immune cells and their products in tumor tissues [17]. The major cell types in the TIME are depicted in Fig. 1 [14, 18–25]. The TIME plays a decisive role in the response of immune checkpoint inhibitors (ICIs) [13, 14]. Some immune cells activate anti-cancer immune responses. For example, patients with higher tumor-infiltrating CD8^+^ T cells have a better response to ICIs [12, 15]. Some immune cells suppress anti-cancer immune responses. For instance, myeloid-derived immunosuppressive cells (MDSCs), a group of immature myeloid cells at different development stages, can coordinate other immune cells and promote immune suppression [25]. Tumor-associated macrophages (TAMs), which are mainly divided into M1-like phenotype and M2-like phenotype, can recruit naïve T cells, regulatory T cells, and T helper 2 cells to inhibit the function of cytotoxic T cells via the M2-like phenotype [26]. Microbes have been found to play a role in the recruitment, differentiation, and proliferation of multiple tumor-infiltrating immune cells.

### The tumor microbe microenvironment

Microbes, such as bacteria, fungi, viruses, and mycoplasmas, reside within tumor tissues [11, 27–31]. The residues of microbes, such as DNA, RNA, peptides, and cell wall components, are observed within cancer cells and tumor-infiltrating immune cells [11]. Some microbial metabolites, including fatty acids and inosine, can accumulate inside tumors and combine with receptors on cancer cells and immune cells [32–35]. Microbe-derived membrane vesicles containing numerous microbial proteins, nucleic acids, and peptidoglycan are universally produced by Gram-positive bacteria [36]. However, their existence within the tumor awaits to be confirmed. All the above components play a role in tumor initiation, progression, metastasis, and immune responses. The microenvironment formed by them distincts from the previously identified tumor microenvironment subtypes and thus can be divided into a new subtype, herein identified as the “tumor microbe microenvironment”. Figure 1 shows the landscape of the tumor microbe microenvironment. The existence and role of viruses in tumors have been reviewed and will not be discussed here in detail [28, 29]. The evidence for intra-tumoral fungi is limited and awaits further elucidation [27, 37]. Emerging studies are revealing the potential roles of intra-tumoral bacteria and therefore will be our focus.

The signatures of the tumor microbe microenvironment are context-dependent and have clinical significance. Firstly, the intra-tumoral microbiome is tumor type-specific and subtype-specific, potentially allowing it to be used as a diagnostic tool [10, 11]. For example, the pancreatic cancer microbiome is dominated by the phylum *Proteobacteria*; while the colorectal cancer microbiome is dominated by the phylum *Firmicutes* and the phylum *Proteobacteria* [11]. Secondly, the microbiome of tumor tissues is significantly distinct from that of normal tissues, as summarized in Table 1, making it a powerful therapeutic target [37–44]. The reasons for some microbes to specifically accumulate within tumor tissues are various. For instance, breast metastasis can express the polysaccharides Gal-GalNAc to bind to the *Fusobacterium nucleatum* that hitchhikes from primary colorectal carcinoma [45, 46]. Some tumors are anaerobic in the center and can attract the anaerobes [47]. The fact that the microbes are attracted by the tumors specifically enables those microbes to be employed as precise anticancer drug vectors. Thirdly, the tumor microbiome is distinct among patients with different survival, making it a potential prognostic tool. In pancreatic cancer, compared with patients with short-term survival, patients with long-term survival have a tumor microbiome with a higher alpha-diversity and a signature of *Pseudoxanthomonas-Streptomyces-Saccharopolyspora-Bacillus clausii* [48].

**Table 1:** Major microbial differences between cancer tissues and non-cancer tissues

Tumor types	Sample	Major microbial differences between tumors and adjacent non-cancer tissues	Major microbial differences between tumors and tissues from healthy people	Reference
Breast cancer	20 tumor tissues and adjacent 20 non-tumor tissues from patients with breast cancer	Increased: *Methylobacterium radiotolerans* Decreased: *Sphingomonas yanoikuyae*		[37]
Breast cancer	Data of 668 breast tumor tissues and 72 adjacent non-tumor tissues from TCGA	Increased: Phylum *Proteobacteria* Decreased: Phylum *Actinomycetes*		[38]
Colorectal cancer	31 tumor mucosal samples and 20 adjacent non-tumor mucosal samples from patients with colorectal cancer, and 30 mucosal samples from healthy controls	Increased: Genera *Lactococcus* and Genera *Fusobacterium* Decreased: Genera Pseudomonas and Genera Escherichia-Shigella	Increased: Phylum *Firmicutes* and Phylum *Fusobacteria* Decreased: Phylum *Proteobacteria*	[39]
Colon cancer	Tumor mucosal samples and adjacent non-tumor mucosal samples from 7 patients with colitis-associated colon cancer (CAC) or 10 patients with sporadic colon cancer (SC), and colon mucosal samples from 10 healthy controls	No significant difference	CAC: Increased: Phylum *Proteobacteria* CAC: Decreased: Phylum *Firmicutes* and Phylum *Bacteroidetes* SC: Increased: Phylum *Fusobacteria* Decreased: Phylum *Bacteroidetes*	[40]
Cervical cancer or intraepithelial neoplasia	30 cervicovaginal swab specimens from patients with invasive squamous cell carcinoma or cervical intraepithelial neoplasia, and 20 normal cervical tissues from healthy controls		Increased: *Gardnerella vaginalis*, *Peptostreptococcus anaerobius*, and *Porphyromonas uenonis* Decreased: *Lactobacillus crispatus*	[41]
Lung cancer	Bronchoalveolar fluid from 20 patients with lung cancer and 8 patients with benign lung mass		Compared with benign lung masses: Increased:Phylum *Firmicutes*, Phylum *TM7*, Genera *Veillonella* and Genera *Megasphaera*	[42]
Lung cancer	143 tumor tissues from patients with lung cancer, 143 adjacent non-tumor tissues from patients with lung cancer, and 33 lung tissues from healthy controls	No significant difference	Increased:Phylum *Proteobacteria* Decreased:Phylum *Firmicutes*	[43]
Thyroid carcinoma	Data of thyroid tumor tissues and adjacent normal tissues from GDC	Decreased: *Micrococcus luteus*, Frankia sp., Anabaena sp. K119, and uncultured Gammaproteobacteria bacterium		[44]

*Abbreviations*: *CAC* Colitis-associated colon cancer, *GDC* The Genomic Data Commons, *SC* Sporadic colon cancer, *TCGA* The Cancer Genome Atlas

The sources of intra-tumoral microbes can be divided into two categories: (I) “aboriginal”, from the tissue type of tumor origin, and (II) “hitchhiker”, from a distant organ or metastasis as depicted in Fig. 1 [49–51]. Gut and oral microbes are important sources of intra-tumoral microbes. A few studies revealed that *Fusobacterium nucleatum* in the oral and *Escherichia coli 17* in the gut could translocate to tumors via the circulation system, indicating a potential influence of the gastrointestinal microbiome on tumor microbiome [50, 52]. The fecal microbiome from patients could partially regulate the intra-tumoral microbiome of mice, which further proved a correlation between gastrointestinal and tumor microbiome [48].

To evaluate the tumor microbiome microenvironment, it is important to select suitable methods of microbiome analysis. Methods of microbiome analysis have been reviewed by others [8, 53]. In the context of the intra-tumoral microbiome, analysis of 16S rDNA sequencing based on short-read sequencing platforms has been the mainstay. However, it is limited in species-level and strain-level resolution, which has a detrimental effect on the identification of functional bacteria. This limitation can be solved by 16S rDNA sequencing depending on full-length sequencing platforms [54]. Metagenomics provides more microbial information than 16S rDNA sequencing [53]. However, its property of being easily interfered by host DNA restricted its application in intra-tumoral microbiome analysis. Some novel microbiome analysis methods, such as machine learning, have a potential role in the diagnosis of microbiome-related diseases and are likely to push forward microbiome researches and clinical transformation in the near future [10]. In addition to analysis methods, the control of confounders is important in microbiome researches. The heterogeneity of different tumor microbiome studies is high, partially caused by undefined confounders. Common confounders include diet, lifestyle, geographical location, and medications [55]. However, their effects on the tumor microbiome are rarely reported and should be considered in future studies. Host genetic alteration has shown a significant correlation with colorectal tumor microbiome, making it a potential confounder in tumor microbiome researches [56].

### The correlation and causality between the tumor microbe microenvironment and the TIME

Multiple cross-sectional studies have observed the correlation between the intra-tumoral microbes and the TIME [57–60]. Previous studies indicated that the immunotherapy efficacy of colorectal cancer was closely related to *Fusobacterium nucleatum*, colibactin-producing *Escherichia coli*, and other carcinogenic microbes [57, 60–64]. Further studies revealed that the *Fusobacterium* DNA load in MSI-high colorectal cancer tumors was inversely associated with tumor-infiltrating FoxP3^+^ T cell density and positively correlated with the ratio of M2-like TAMs to TAMs. The *Fusobacterium* load had no significant correlation with tumor-infiltrating CD3^+^, CD4^+^, and CD8^+^T cells, suggesting that *Fusobacterium nucleatum* promoted the tumor progression mainly through the expansion of suppressive immune cells [65]. Paradoxically, another study found that the *Fusobacterium nucleatum* DNA load in colorectal cancer was negatively correlated with the density of stromal CD3^+^ T cells, especially CD3^+^CD4^+^CD45RO^+^ subgroups. However, *Fusobacterium nucleatum* DNA load had no significant correlation with FoxP3^+^ T cells or TAMs [58]. The negative correlation between the *Fusobacterium* load and CD3^+^T cell or CD4^+^T cell density was supported by other studies [57, 59]. Aside from *Fusobacterium nucleatum*, *Escherichia coli* load was negatively correlated with the density of colorectal tumor-infiltrating CD3^+^ T cells [60]. In gastric cancer, the abundance of genus *Stenotrophomonas* and genus *Selenomonas* in the gastric mucosa was positively correlated with the density of tumor-infiltrating BDCA2^+^pDCs and Foxp3^+^Tregs [66]. Overall, there is a correlation between the abundance of specific microbes and the density of anticancer T cells or suppressive immune cells.

Administrating specific microbes into germ-free mice and detecting the TIME changes are crucial steps in determining the causality between the tumor microbe microenvironment and the TIME. By using a mouse model with breast cancer, Lishay Parhi and colleagues found that the *Fusobacterium nucleatum* specifically accumulated inside the tumors and reduced tumor-infiltrating CD4^+^ and CD8^+^ T cells. The researchers ruled out the influences of cancer cell proliferation or apoptosis, proving that the *Fusobacterium nucleatum-*mediated TIME changes were the major contributors to tumor growth [46]. By using the mouse model with APC^min/+^ colorectal carcinoma, Aleksandar D. Kostic and colleagues suggested that *Fusobacterium nucleatum* recruited CD11b^+^ myeloid cells to the TIME [67]. Chengcheng Jin and colleagues established sterile mouse models with lung cancers and discovered that transplanted bacteria stimulated myeloid cells to secrete IL-23. The research team also found that the bacteria induced γδ T cell to proliferate and secrete IL-17 [68]. Similar results were reported in the mice with colorectal carcinoma [69]. Overall, these findings suggest that specific microbes can change the TIME.

### Mechanisms underlying the role of the tumor microbe microenvironment in the TIME

**Fig. 2:**
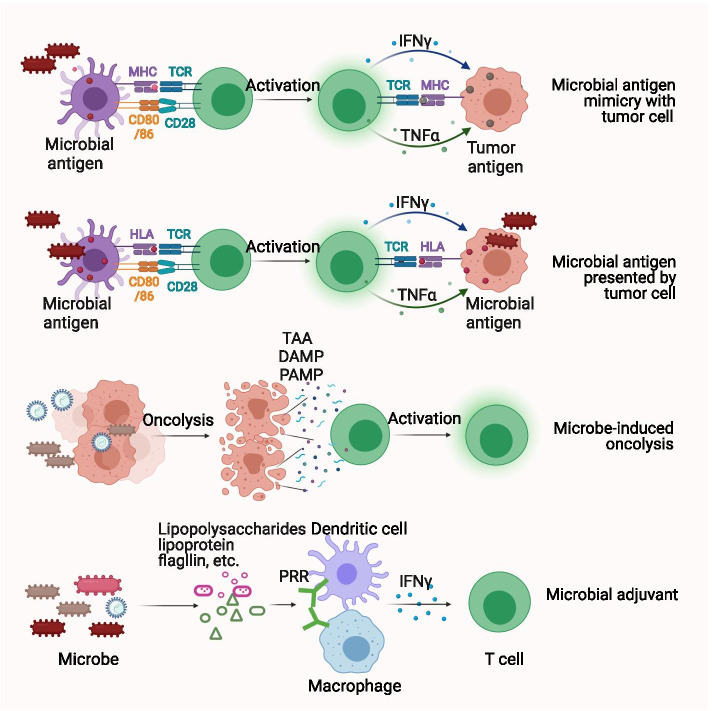
Intra-tumoral microbes provide antigenicity and adjuvanticity to promote inflammatory tumor immune microenvironment. Microbial antigens can be presented by HLA molecules on both tumor cells and tumor-infiltrating immune cells, informing their potential to elicit anti-tumoral response. Microbe antigens share similar antigen epitopes with tumor antigens and elicit microbe-specific T cells that can recognize and kill tumor cells. Microbes lyse tumor cells to release TAA, DAMP, and PAMP to elicit inflammatory tumor immune microenvironment. Also, microbe themselves serve as immunostimulatory adjuvants to further promote inflammatory tumor immune microenvironment. Abbreviations: CD = cluster of differentiation, DAMP = damage-associated molecular pattern, HLA = human leukocyte antigen, IFNγ = interferon-gamma, MHC = major histocompatibility complex, PAMP = pathogen-associated molecular pattern, PRR = pattern recognition receptor, TAA = tumor-associated antigen, TCR = T-cell receptor, TNFα = tumor necrosis factor-alpha

**Fig. 3:**
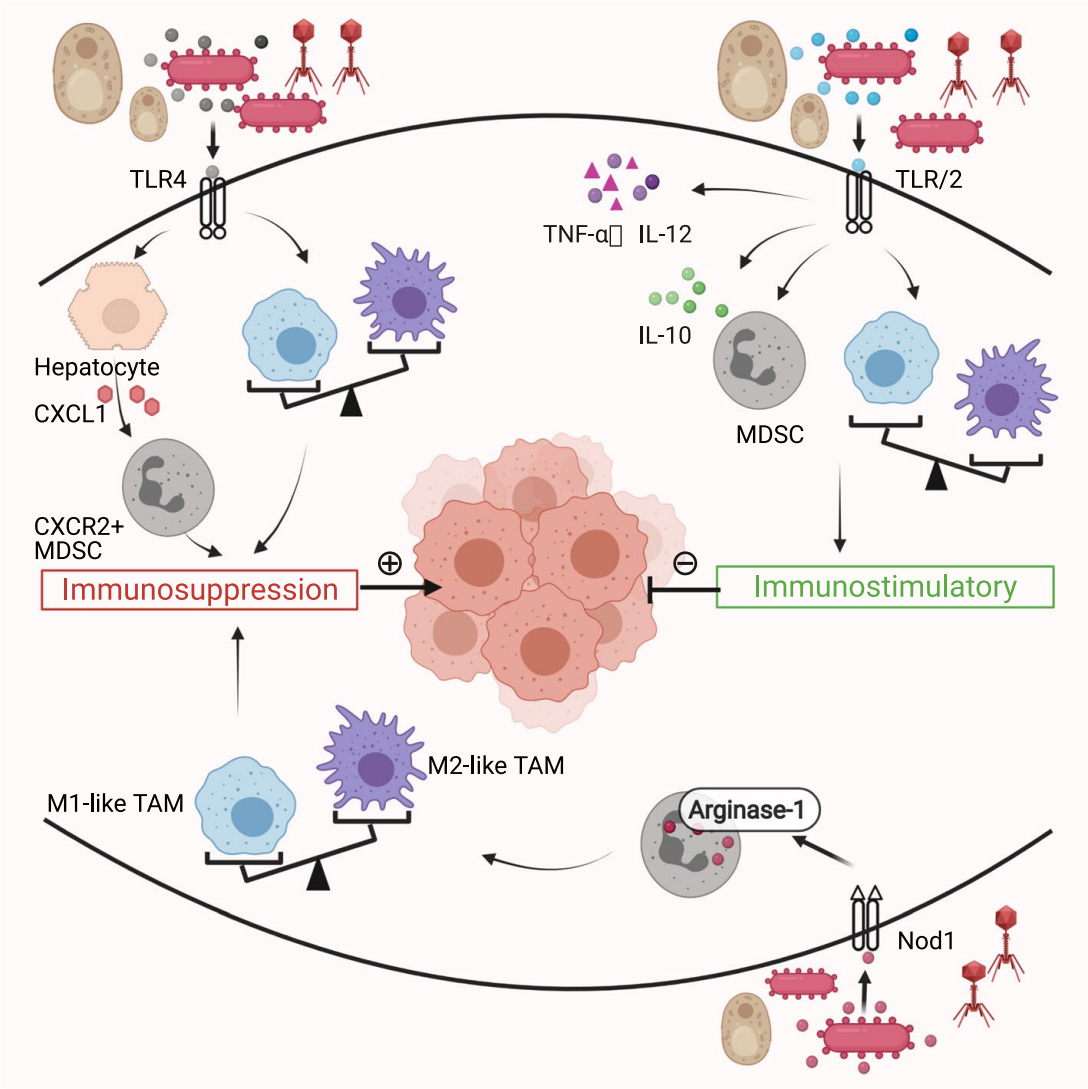
Microbial immunomodulation mediated by pattern recognition receptors in the tumor microenvironment. TLRs and NLRs are the major subtypes of pattern recognition receptors. Some microbes activate TLR4 to promote MDSCs infiltration and M2-like TAM polarization, resulting in an immunosuppressive tumor microenvironment. On the contrary, some microbes activate TLR2 to promote M1-like TAM polarization and suppress MDSCs function. TLR agonist synergizes with interferon-γ to increase pro-inflammatory cytokines TNF-α, IL-12 and decrease IL-10, forming an inflammatory tumor microenvironment. Nod1, a member of the NLRs family, promotes MDSCs proliferation and arginase-1 expression and thereby leading to M2-like TAM repolarization. Abbreviations: CXCL = The chemokine (C-X-C motif) ligand, CXCR = The chemokine (C-X-C motif) receptor, MDSC = myeloid-derived immunosuppressive cell, NLRs = Nucleotide-binding domain and leucine-rich repeat–containing receptors, Nod = Nucleotide-binding domain, TAM = tumor-associated macrophage, TLR = Toll-like receptor, TNF = tumor necrosis factor

**Fig. 4:**
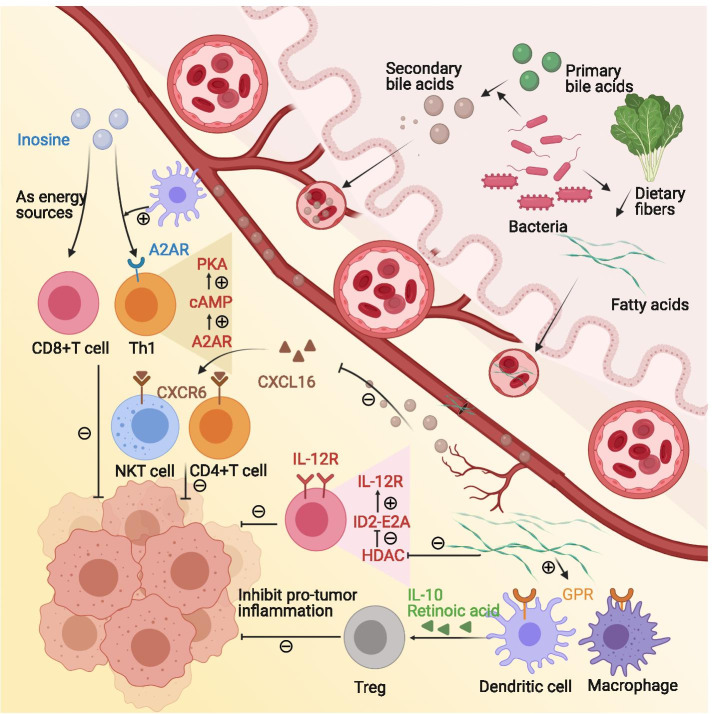
Microbe-derived metabolites modulate the tumor immune microenvironment. Microbial metabolites, such as short-chain fatty acids, bile acids, and inosine, can enter to blood and modulate the tumor immune microenvironment. Butyric acid, a member of short-chain fatty acids, increases the level of IL-10 and retinoic acid in the intestinal microenvironment, which promotes regulatory T cells differentiation and proliferation. Butyrate-mediated HDACs inhibition leads to up-regulation of the transcriptional regulator ID2 and thus upgrading the IL-12R signaling pathway in CD8^+^ T cells. Secondary bile acids are produced by gut microorganisms from primary bile acids. ω-murocholic acid, a member of secondary bile acids, down-regulates CXCL16 secretion and reduces natural killer T cells and CD4^+^ T cells recruitment. Inosine binds to A2AR on T cells and initiates the inosine-A2AR-cAMP-PKA signaling pathway. With the costimulatory effects from the dendritic cells, inosine induces naïve T cells to differentiate into Th1. Besides, Inosine is alternative energy of glucose in cytotoxic T cells. Abbreviations: A2AR = adenosine 2A receptor, CXCL = the chemokine (C-X-C motif) ligand, CXCR = the chemokine (C-X-C motif) receptor, HDAC = histone deacetylase, GPR = G protein-coupled receptor, Th1 = helper 1 T cell

**Fig. 5:**
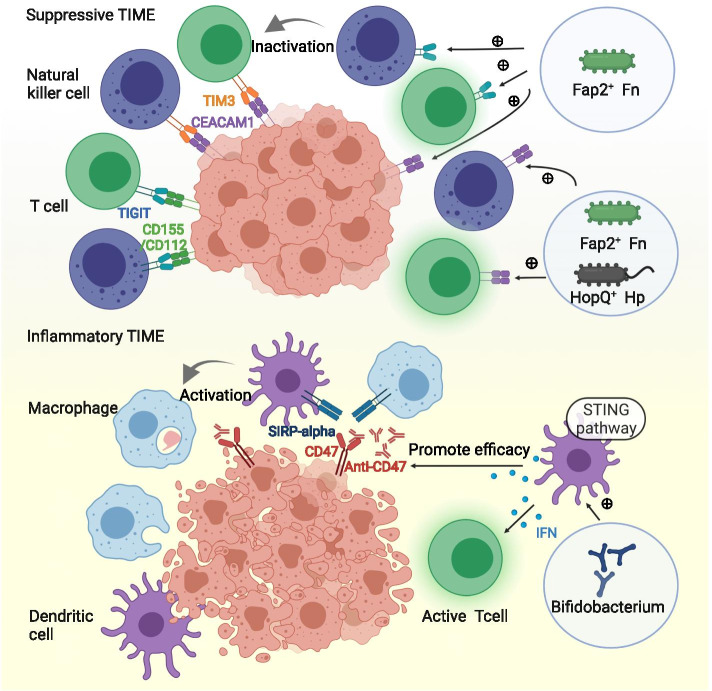
Microbial stimulation of inhibitory checkpoints modulates the tumor immune microenvironment. *Fusobacterium nucleatum* inhibits the activity of natural killer cells and cytotoxic T cells via interaction between Fap2 and TIGIT or CEACAM1. *Helicobacter pylori* acts on CEACAM1 through its outer membrane protein HopQ protein to inhibit immune cells. *Bifidobacterium* upregulates the expression of IFN-I in dendritic cells through the STING signaling pathway, thereby promoting antigen cross-presentation and T cell activation to enhance the efficacy of CD47 blockade. Abbreviations: STING = the stimulator of interferon gene

To explore the causal relationship between the tumor microbe microenvironment and the TIME, we need to further explore the mechanisms underlying the changes outlined above. The mechanisms claimed by recent studies include: (I) The presentation of bacterial peptides by cancer cells and immune cells, (II) bacterial antigen mimicry with tumor antigens, (III) microbe-induced immunogenic cell death, as depicted in Fig. 2, (IV) adjuvants and pattern recognition receptor-mediated signaling pathway, as depicted in Fig. 3, (V) microbe-derived metabolites, as depicted in Fig. 4, and (VI) stimulation of inhibitory checkpoints, as depicted in Fig. 5. Understanding the mechanisms underlying microbial effects in the TIME will bring new insight into new drug researches.

#### Microbial antigen presented by cancer cells potentially activates antitumor T cells

The successful elicitation of adaptive antitumor responses requires two key steps. The first step is that tumor antigens are presented by human leukocyte antigen (HLA) to activate CD8^+^ T cells. The second step is the recognition and killing of antigen-presenting cancer cells by the activated CD8^+^ T cells. However, cancer cells can hide their antigens from immune cells through multiple mechanisms [70]. Bacterial peptides are prevalent among melanoma metastasis [71]. They can be presented by HLA molecules on both melanoma cells and tumor-infiltrating immune cells, demonstrating their potential to act as tumor-specific antigens. B cells loaded with the bacterial peptides-HLA complex can activate tumor-infiltrating T cells to secrete interferons in vivo, further indicating their immunogenic features. Several bacterial peptides are prevalent among multiple melanoma lesions of the same patients. Such peptides can be developed for personalized medication. Other bacterial peptides are prevalent across multiple melanoma patients, which suggests their potential as generalizable microbial targets [71]. Given the fact that bacterial peptides are exogenous, they are more likely to trigger an immune response compared to tumor antigens [72].

However, questions remain which still need answers. First of all, the study did not detect the presentation of bacterial peptides in adjacent normal tissues. If the bacterial peptides also occurred in normal tissues, these tissues would inevitably be attacked when applying microbial antigen-based therapies. Future studies should try to identify bacterial peptides that are expressed differently in tumors and normal tissues. Secondly, the bacterial peptides presented by cancer cells did not trigger effective anti-cancer immunity in vivo and the underlying reasons are unclear. The interaction between bacterial peptides, cancer cells, and immune cells in vivo needs further investigation.

#### Microbial antigen mimicry shared with tumor antigens activates antitumor T cells

Antigen mimicry is a phenomenon in which microbes share similar antigen epitopes with tumor antigens. Microbe-specific T cells recognize and kill cancer cells that express similar antigen epitopes, a process called cross-reactivity [73].

To determine whether antigen mimicry is a common mechanism for microbes to affect anti-tumor immunity, researchers need to identify cross-reactive antigen epitopes and the epitope-specific T cell. Alexandra Snyder and colleagues analyzed the tumor neoantigen epitopes in melanoma patients with different prognoses. They found that some tumor neoantigen epitopes were homologous with microbial epitopes. Higher homology was associated with a better clinical prognosis [74]. This finding suggests that antigen mimicry exists in tumors and has the potential to impact the immune response. Aurélie Fluckiger and colleagues detected T cells that cross-recognized tumor antigens and microbial antigens in cancer patients. They also identified a bacteriophage antigen called tape measure protein that had a similar epitope to the tumor-associated antigen PSMB4. The tape measure protein was presented by the MHC-I in a mouse model to generate specific CD8^+^ T cells. These CD8^+^ T cells recognized and attacked tumor cells expressing PSMB4, thereby improving PD-1 blockade efficacy and prolonging the survival of the mice [75]. *Bifidobacterium breve* with epitope SVY could induce the production of SVY-specific T cells. These T cells recognized and attacked melanoma cells expressing epitope SIY. Eliminating *Bifidobacterium breve* promoted tumor growth [76]. Shin-Heng Chiou and colleagues analyzed more than 770,000 T-cell receptor sequences from 178 lung cancer patients. They found that compared with normal tissues, tumor tissues overexpressed a protein that cross-reacted with Epstein-Barr virus and *E. coli*. They suggested that cross-reactions existed in multiple samples of lung cancer [77]. In the future, it is necessary to explore the presence of antigen mimicry in various types of tumors and the intensity of the anti-tumor immune response that it triggers. Naturally existing or artificially designed “mimic antigens” will provide a novel perspective for cancer treatment.

#### Microbe-induced immunogenic cell death promotes inflammatory TIME

Immunogenic cell death (ICD) is a form of cell death in which dead cells release antigens and adjuvants to enhance immune responses. It can be triggered by microbes. Some researchers combined the empty envelopes of bacteria and drug oxaliplatin to treat murine models with advanced colorectal cancer. The combination strategy strongly suppressed tumor growth and prolonged the survival of murine models via strengthened ICD [78]. Oncolytic viruses or bacteria specifically orient to the tumor microenvironment and lyse tumor cells, thereby releasing tumor antigens, damage-associated molecular patterns, and pathogen-associated molecular patterns to recruit peripheral immune cells to the TIME or reboot pre-existing antitumor immune cells in the TIME [79, 80]. Simultaneously, microbes themselves serve as promising immune adjuvants to promote inflammatory TIME, which further boosts antitumor immunity. The adjuvanticity of microbes and underlying mechanisms are discussed in detail in the next paragraph.

#### Microbial adjuvanticity mediated by pattern recognition receptors modulates the TIME

Microbial adjuvanticity refers to the immunomodulatory effects of the pathogen-associated molecular patterns that are derived from microbes. Pathogen-associated molecular patterns can be sensed by pattern recognition receptors (PRRs)—a key step for the microbes to elicit an innate immune response and subsequent adaptive immune response [73, 81]. Toll-like receptors (TLRs) are the most studied subtype of PRRs [82]. Microbial activation of TLRs roles as a double-edged sword in the tumor immune microenvironment.

Firstly, intra-tumoral microbes drive the formation of immunosuppressive tumor microenvironment through TLRs. In murine models with the cancerous pancreas, the intra-tumoral microbes selectively activated TLRs in monocytic cells to induce M2-like TAM differentiation. Clearance of microbes via antibiotics significantly promoted T cell activation, M1-like TAMs differentiation, and PD-1 up-regulation, while at the same time decreased MDSCs and M2-like TAMs inside the tumor [83]. Bacterial lipopolysaccharides recognized by TLR4 induced hepatocytes to express CXCL1. CXCL1 was a chemokine that recruited CXCR2^+^ polymorphonuclear MDSCs to form an immunosuppressive environment, promoting cholangiocarcinoma in mice [84]. Similarly, *Fusobacterium* recognized by TLR4 upregulated the IL-6/p-STAT3/c-MYC signaling pathway, leading to M2-like TAM polarization and colorectal carcinoma progression [85].

Secondly, intra-tumoral microbes maintain the immunostimulatory tumor microenvironment through TLRs, therefore acting as cancer-fighting agents. In lung cancer murine models, bacterial lipoproteins activated TLR2. As a result, MDSCs were reprogrammed to differentiate into inflammatory M1 phenotypes. The immunosuppressive functions of MDSCs were blocked [86]. TLR agonist synergized with interferon-γ to increase pro-inflammatory cytokines TNF-α, IL-12p40, and IL-12p70 and decrease IL-10, forming an inflammatory microenvironment to activate anti-tumor immune response [87]. It is noteworthy that not all inflammation is beneficial. Some chronic inflammation is tumorigenesis and can be induced or suppressed by the interaction between microbes and TLRs. For instance, TLR-5 sensed bacterial flagellin to upregulate a protein called high mobility group box 1, which triggered inflammation and skin cancer initiation [88]. *Lactobacillus* species triggered IL-10 expression to inhibit colon inflammation in a TLR-6 dependent way. Inflammation-induced colorectal cancer was prevented as a result [89].

Nucleotide-binding domain and leucine-rich-repeat–containing receptors (NLRs) are another group of PRRs that show increasing significance in the interaction between host immunity and microbes [90]. Nucleotide-binding domain 1 (Nod1), a member of the NLRs family, is a cytosolic protein expressed in various cells and functions as a sensor for microbial peptidoglycan fragments. Activation of Nod1 promoted MDSCs to proliferate and express arginase-1. Arginase-1 sustained the immunosuppressive potential of MDSCs and promoted the M2-like repolarization of macrophages. An immunosuppressive tumor microenvironment was formed as a result [91].

Overall, microbes modulate the tumor immune microenvironment via PRRs. Drugs targeting at PRRs can be adjuvants to checkpoints blockade. Notably, PRRs encompass numerous sensors or receptors, some of which are recently discovered and await further investigation [81]. A specific microbe can interact with various PRRs simultaneously. Therefore, the immunomodulatory effect of microbes is the sum of many different PRRs-mediated signaling pathways.

#### Microbe-derived metabolites modulate the TIME

Microbial metabolites such as short-chain fatty acids (SCFAs), bile acids, and inosine can enter the blood. Receptors of some microbe-derived metabolites are expressed on cancer cells and tumor-infiltrated immune cells, indicating the potential role of microbe-derived metabolites in the tumor microenvironment.

SCFAs are the products of dietary fibers fermented by intestinal anaerobic bacteria. SCFAs include acetic acid, propionic acid, and butyric acid. For normal intestinal epithelial cells, SCFAs function as an inhibitor of pro-tumor inflammation. For example, butyric acid was recognized by G protein-coupled receptors (GPRs) on the surface of colon cells and immune cells. In this way, butyric acid increased the level of IL-10 and retinoic acid in the intestinal microenvironment, which promoted the differentiation of naïve T cells into regulatory T cells. It also promoted the proliferation of regulatory T cells, thereby suppressing pro-tumor inflammation [92]. For tumor tissues, SCFAs accumulate within tumors, regulating tumor proliferation and the tumor microenvironment. For instance, butyric acid inhibited histone deacetylases (HDACs) in a GPR-independent manner [32] [93]. HDACs are chromatin regulatory factors that are expressed abnormally in a variety of human cancers and can result in aberrant chromatin modification. Selective HDAC8 inhibitors enhanced the expression of CCL4, a key chemokine for T cell migration. This process increased the density of CD8^+^ T cells in mice with hepatic carcinoma. In addition, there was a decrease in the density of Treg cells in an unclarified manner. These changes in the tumor microenvironment promoted anti-tumor immunity and enhanced PD-L1 blockade efficacy in mouse models [94]. In melanoma, HDACs inhibitors up-regulated PD-1/PD-L1 and suppressed CD4^+^ T cells apoptosis, which further proved the potency of HDAC inhibition in tumor immune microenvironment modulation [95, 96]. Butyrate-mediated HDACs inhibition leads to up-regulation of the transcriptional regulator ID2 in the nucleus. ID2 bound to the transcription factor E2A and relieved its inhibitory effect on IL-12 receptor expression, significantly upregulating the IL-12R signaling pathway in CD8^+^ T cells. This process increased CD8 ^+^ T cell density and activation in the tumor immune microenvironment [93]. It was consistent with the previous finding in which butyrate promoted (i) the differentiation of naïve cells into cytotoxic T cells, and (ii) the secretion of IFN-γ and granzyme B through HDACs inhibition [97]. Preclinically, butyric acid improved the efficacy of immunogenic drugs oxaliplatin and PD-L1 blockade in mice. Clinically, serum butyrate levels were positively correlated with oxaliplatin response in patients [93]. However, contradictory results occurred in mice and patients treated with CTLA-4 blockade, in which high systemic butyrate and propionate levels were associated with poor outcomes. This process was associated with increased Treg cells and decreased antitumor T cells [98]. Overall, SCFAs are potential therapeutic targets for immunotherapy. The role of SCFAs is context-dependent and needs to be further clarified before translation into clinical practice.

Secondary bile acids are produced by gut microbes from primary bile acids [99]. Secondary bile acids, such as ω-murocholic acid, down-regulate the secretion of chemokine CXCL16 of hepatic sinusoidal endothelial cells. Natural killer T cells recruited by CXCR6-CXCL16 interaction are reduced as a result. Antibiotics can eliminate microbes and reverse the above effects [100]. CXCR6 agonists can promote the elimination of senescent liver cells by natural killer cells and CD4^+^ T cells, thus reducing the risk of hepatic carcinogenesis [101]. Overall, the current study indicates that the regulation of natural killer cells by bile acids through CXCL16-CXCR6 plays an important role in the initiation and progression of hepatic cancer. More studies are needed to reveal the impact of bile acids on other immune cells.

Inosine is a purine metabolite derived from *Bifidobacterium pseudolongum*. It binds to the adenosine 2A receptor (A2AR) on T cells and initiates the inosine-A2AR-cAMP-PKA signaling pathway. This is followed by the phosphorylation of a protein called cAMP response element-binding protein. With the costimulatory effects from dendritic cells, inosine induces naïve T cells to differentiate into CD4^+^Th1 [33]. Compared with the PD-L1 blockade alone, the combination of inosine with the PD-L1 blockade increases CD8^+^ T cells infiltration and IFN-γ secretion in the TIME [34]. It should be noted that inosine is used in effector T cells as an alternative energy source in place of glucose. Cancer cells cannot utilize inosine, making it an ideal fuel which motivates T cells to kill cancer cells [35]. Some other bacteria-derived metabolites, such as N-acetylmuramic acid and N-acetylglucosamine, have an obvious immunosuppressive effect [102]. Their roles in the tumor immune microenvironment await further clarification.

#### Microbial stimulation of inhibitory checkpoints modulates the TIME

Immune checkpoints work by inactivating immune cells. Inhibitory checkpoints include PD-1, CTLA-4, TIM-3, LAG-3, TIGIT, and CEACAM1. *Fusobacterium nucleatum* inhibited the activity of natural killer cells and cytotoxic T cells through interaction between Fap2 and TIGIT, or interaction between Fap2 and CEACAM1 [103–105]. *Helicobacter pylori* acted on CEACAM1 through its outer membrane protein, HopQ, to inhibit immune cells [106]. Interaction between HopQ and CEACAM1 mediated the translocation of virulence factor CagA and the release of IL-8. Through this process, *Helicobacter pylori* promoted gastric epithelial damage, inflammation, and tumorigenesis [107]. Apart from *Helicobacter pylori* and *Fusobacterium nucleatum*, other bacteria, such as pathogenic *Neisseria*, can also bind to CEACAM1. Therefore, the interactions between microbes and immune checkpoints are not uncommon and thus deserve more exploration.

An additional checkpoint, CD47, is expressed on the surface of tumor cells. SIRPα is the ligand of CD47 and is expressed on dendritic cells and macrophages. CD47-SIRPα interaction inhibits antigen presentation and phagocytosis. Multiple antagonists targeting the CD47-SIRPα signaling have been under development [108]. *Bifidobacterium* intravenously injected into mice gathered in the tumor site. It upregulated the expression of IFN-I in dendritic cells through the stimulator of the interferon gene (STING). IFN-I is a critical cytokine for antigen cross-presentation and T cell activation. Intratumor injection of antibiotics cleared *Bifidobacterium* and reduced the efficacy of CD47 blockade, suggesting that *Bifidobacterium* could be a potential adjuvant for CD47 blockade [109].

### Modulation of the tumor microbe microenvironment as a combination for immune checkpoint inhibitors

**Fig. 6:**
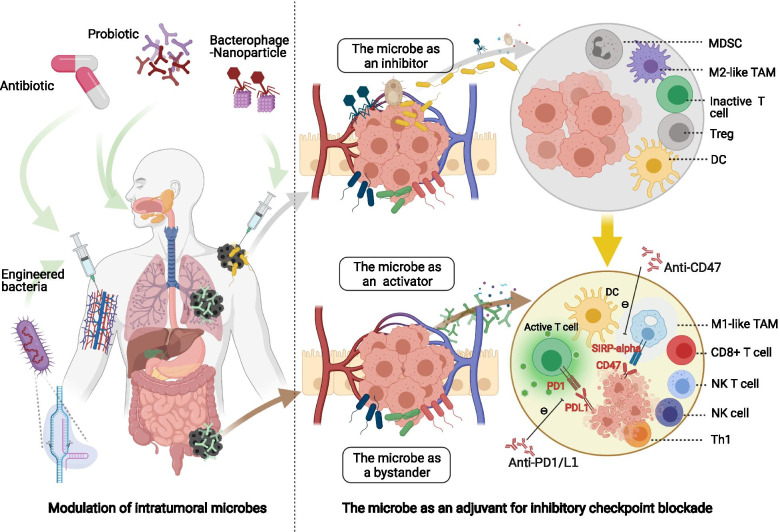
Modulation of the tumor microbe microenvironment acts as a combination for immune checkpoint inhibitors. The modulation strategies of the tumor microbe microenvironment include antibiotic, probiotic and synthetic biology. Microbes can role as an immune inhibitor, activator, or bystander. Adding immunostimulatory microbes or clearance of immunosuppressive microbes can enhance the efficacy of immune checkpoint inhibitors

The successful induction of anti-tumor adaptive immune response requires three elements: antigen, adjuvant, and suitable immune microenvironment. The tumor microbe environment impacts these three elements simultaneously, making it a promising combination for ICIs [110, 111]. Clinic strategies for endogenous microbial modulation include antibiotics and probiotics. Exogenous microbial regulation utilizes microbes developed by synthetic biology methods, such as engineered bacteria and oncolytic viruses. The use of *Bacille Calmette-Guérin* (BCG) for treating non-muscle-invasive bladder cancer as well as the use of oncolytic virus talimogene laherparepvec (T-VEC) for treating advanced melanoma are two examples of successful tumor microbe microenvironment regulation [112–115]. Emerging evidence of other modulation strategies is shown in Fig. 6 and will be discussed below in detail.

#### Synthetic biology and immune checkpoint inhibitors

Microbes have been employed as programmable drug-delivery platforms for a long time [116, 117]. Recently, microbes have been designed to potentiate immunotherapy. A non-pathogenic *Escherichia coli* was engineered to load CD47 nanobody blockade. The strain colonized and released CD47 nanobody blockade within tumors. It then activated tumor-infiltrating T cells to eliminate tumors. Tumor regression was also observed in noninjected metastasis. Another benefit was that select adverse effects that occurred with CD47 blockade were less frequent [118]. A similar strategy was utilized in the development of an engineered *Escherichia coli* strain called SYNB1891. The strain activated the STING pathway in antigen-presenting cells and thereby enhanced the phagocytosis of cancer cells [119]. Aside from adding immunostimulatory microbes into tumors, synthetic biology can remove immunosuppressive microbes away from tumors. For, example, the complex of *Fusobacterium nucleatum*-specific bacteriophages and nanosilver particles cleared pro-tumoral *Fusobacterium nucleatum*, reduced intra-tumoral myeloid-derived suppressive cells, and enhanced ICIs efficacy [120]. Overall, Synthetic biology is a potential combination strategy for immunotherapy. Checkpoint blockades can be delivered to the tumor site by engineered microbes, augmenting efficacy and reducing systemic toxicity.

#### Antibiotics and immune checkpoint inhibitors

Clinically, antibiotics administration negatively correlates with clinical outcomes of ICIs treatment [121, 122]. In a multicenter retrospective study including patients with non-small cell lung cancer by Alessio Cortellini and colleagues, prior antibiotic exposure negatively correlated with the outcomes of first-line immunotherapy instead of first-line chemotherapy. And it indicated the immunomodulatory effects of antibiotics [123]. In contrast, in another study by Alessio Cortellini and colleagues, prior antibiotic exposure did not affect the outcomes of patients receiving chemo-immunotherapy [124]. The contrast results of antibiotics on immunotherapy alone and chemo-immunotherapy could be explained by antibiotic-mediated microbial regulation. On the one hand, antibiotics can cause microbial disturbance to impair ICIs efficacy. On the other hand, antibiotics can clear up chemotherapy-induced detrimental microbes to enhance ICIs efficacy. And these two effects cancel out. Monitoring of microbial changes is needed in both preclinical experiments and clinical trials to confirm the role of microbes in the interaction between antibiotics and ICIs.

Notably, systemic administration of antibiotics can cause flora disturbance in both the gut and tumor tissues [125]. It is difficult to distinguish whether the impact of antibiotics on immunotherapy is mediated by intra-tumoral microbes or gut commensals. Intra-tumoral administration of antibiotics is more likely to have a precise modulation effect, acting more exclusively on intra-tumoral microbes. For this reason, intra-tumoral administration should be employed in preclinical models to explore microbial effects on the TIME.

#### Probiotics and immune checkpoint inhibitors

Several clinical trials combining probiotics and ICIs are ongoing, as listed in Table 2. Oral probiotics have been found to restore anti-cancer immunity and ICIs efficacy by pleiotropic mechanisms [126]. However, changes in the tumor microbe microenvironment brought on by oral probiotics have not been characterized and thus need to be detected in future clinical trials. One preclinical study claimed that *Bifidobacterium* accumulated in the tumor microenvironment after high dose oral administration [109]. Another preclinic study reported that intra-urethral administrated microbe CP1 colonized prostate tumors specifically. CP1 was a patient-derived commensal. It increased the extent of immunogenic cell death and the density of anti-cancer immune cells inside the tumor. It also provided a powerful therapeutic strategy that turned an immunologically cold tumor into a hot one [127].

**Table 2:** Clinical trials of the probiotic and immune checkpoint blockade combination registered on NIH ClinicalTrials.gov

NCT number	Probiotic	Immune checkpoint inhibitor	Disease	Design
NCT03775850	EDP1503	Pembrolizumab	MSS colorectal cancer, triple negative breast cancer, and PD-1 blockade relapsed cancer	Phase I/II, open-label, triple-cohort, non-randomized
NCT03595683	EDP1503	Pembrolizumab	Advanced melanoma naïve or refractory to PD-1 blockade	Phase II, dual-cohort, non-randomized
NCT03637803	MRx0518	Pembrolizumab	Advanced and/or metastatic or recurrent cancer, including NSCLC, renal cell carcinoma, bladder cancer, and melanoma	Phase I/II, open-label, one-cohort non-randomized
NCT04208958	VE800	Nivolumab	Advanced or metastatic cancer, including melanoma, gastric cancer, gastroesophageal junction adenocarcinoma, and MSS colorectal cancer	Phase I/II, multicenter, open-label, one-cohort, non-randomized
NCT03817125	SER-401	Nivolumab	PD-1 blockade naive, unresectable or metastatic melanoma	Phase Ib, multicenter, randomized, placebo-controlled, blinded
NCT04601402	GEN-001	Avelumab	Locally advanced or metastatic solid tumors which have progressed on PD-1/PD-L1 blockade, including NSCLC, squamous cell carcinoma of head and neck, and urothelial carcinoma	Phase I/Ib, dual-cohort, non-randomized

*Abbreviations*: *MSS* Microsatellite stable, *NSCLC* Nonsmall-cell lung cancer, *PD-1* Programmed cell death protein-1, *PD-L1* Programmed cell death protein-ligand 1

## Conclusions

Overall, Microbes, including bacteria, fungus, viruses, and their components and metabolites, inhabit various tumor tissues, herein defined as the tumor microbe microenvironment. Current studies have revealed the role of some microbes as immune activators, inhibitors, or bystanders. Considering the multifaceted roles of the tumor microbe microenvironment, its modulation strategies including synthetic biology, antibiotics, and probiotics can be potential combinations for immunotherapy.

Some phenomena or questions remain to be further elucidated, including the relationship among oral, gut and tumor microbiome. The selection of microbiome analysis methods and identification of possible confounders need to be considered carefully in microbiome researches.

The tumor microbe microenvironment is a novel field facing major challenges and chances. Achieving a comprehensive understanding of intra-tumoral microbes and their roles in the tumor immune microenvironment will provide a conceptual shift toward studying the cancer-immune-microbial relationship. Tumor microbiome may have the potential to be used as a prognostic or predictive tool. It could also be helpful for new anti-cancer drug development. Importantly, it may unlock the next wave of precision medicine and immunotherapy combination strategies.

## Data Availability

Not applicable.

## Abbreviations

A2AR: Adenosine 2A receptor
CD: Cluster of differentiation
CXCL: The chemokine (C-X-C motif) ligand
CXCR: The chemokine (C-X-C motif) receptor
DAMP: Damage-associated molecular pattern
GPR: G protein-coupled receptor
HDAC: Histone deacetylase
HLA: Human leukocyte antigen
ICIs: Inhibitory checkpoint inhibitors
IFNγ: Interferon gamma
MDSC: Myeloid-derived immunosuppressive cell
MHC: Major histocompatibility complex
Nod: Nucleotide-binding domain
NLRs: Nucleotide-binding domain and leucine-rich repeat–containing receptors
PRRs: Pattern recognition receptors
TAA: Tumor associated antigen
TAM: Tumor-associated macrophage
TCR: T-cell receptor
Th1: Helper 1 T cell
TIME: Tumor immune microenvironment
TLRs: Toll-like receptors
TNF: Tumor necrosis factor
